# Clinical application of next-generation sequencing for Mendelian diseases

**DOI:** 10.1186/s40246-015-0031-5

**Published:** 2015-06-16

**Authors:** Saumya Shekhar Jamuar, Ene-Choo Tan

**Affiliations:** Genetics Service, Department of Paediatrics, KK Women’s and Children’s Hospital, Singapore, Singapore; Paediatrics Academic Clinical Programme, SingHealth Duke-NUS Graduate Medical School, Singapore, Singapore; KK Research Centre, KK Women’s and Children’s Hospital, 100 Bukit Timah Road, 229899 Singapore, Singapore

**Keywords:** Next-generation sequencing, Whole exome sequencing, Clinical applications, Mendelian diseases

## Abstract

Over the past decade, next-generation sequencing (NGS) has led to an exponential increase in our understanding of the genetic basis of Mendelian diseases. NGS allows for the analysis of multiple regions of the genome in one single reaction and has been shown to be a cost-effective and efficient tool in investigating patients with Mendelian diseases. More recently, NGS has been successfully deployed in the clinics, with a reported diagnostic yield of ~25 %. However, recommendations on clinical implementation of NGS are still evolving with numerous key challenges that impede the widespread use of genetics in everyday medicine. These challenges include when to order, on whom to order, what type of test to order, and how to interpret and communicate the results, including incidental findings, to the patient and family. In this review, we discuss these challenges and suggest guidelines on implementing NGS in the routine clinical workflow.

## Introduction

Mendelian diseases, also known as monogenic diseases, are disorders caused by mutations in one gene and include diseases like thalassemia, cystic fibrosis, among others. Mendelian diseases are considered to be rare individually but collectively occur at a rate of 40 to 82 per 1000 live births, with an estimated 7.9 million children being born annually with a serious birth defect of genetic or partially genetic origin [1, 2]. These disorders tend to run in families, although it has been found that a significant number are caused by de novo events [3]. Depending on the pathomechanism, the phenotype is manifested in a dominant (where one allele is mutated) or recessive (where both alleles are mutated) manner [4]. Of the estimated 20,000–25,000 protein-coding genes in the human genome, mutations in 3348 genes have been associated with Mendelian diseases [5].

Sanger sequencing, also known as the dideoxy method [6], has been the gold standard in molecular diagnostics in Mendelian diseases and remains the test of choice for clinical genetic testing; the purpose of which is to confirm a suspected diagnosis and allow more accurate genetic counseling. However, Sanger sequencing can only analyze one DNA segment at a time and is thus laborious and time consuming. For diseases with genetic heterogeneity, like retinitis pigmentosa, cardiomyopathy, and deafness, a gene-by-gene Sanger sequencing approach has not been shown to be economical or efficient [7].

Recent advances over the past decade have allowed for high-throughput sequencing, and these advances are collectively referred to as next-generation sequencing (NGS). NGS has allowed for substantial increase in sequencing content while dramatically reducing the cost of sequencing per base. This allows for simultaneous interrogation of multiple genes through one single reaction and has been proven to be an effective alternative for establishing the genetic basic of Mendelian diseases in the research setting [8–11], and more recently, in the clinical setting [12–16].

In this review, we shall aim to discuss the clinical utility of NGS, including the opportunities and challenges that arise from the clinical standpoint. We will also focus our discussion on ways of implementing NGS in the routine clinical workflow.

### Next-generation sequencing: brief overview

The process of NGS starts with extraction of DNA of an individual, most commonly from peripheral leukocytes obtained from blood sample but can be from another tissue such as buccal swab or saliva. The DNA is then broken down into short fragments and amplified using polymerase chain reaction (PCR) or hybridization-based approaches. The regions that are amplified could include either a subset of genes (targeted approach) or all the genes in the genome [9]. When sequencing all the genes, if only the protein-coding regions are amplified, the method is referred to as whole exome sequencing (WES). However, if the target is the entire genome, then it is known as whole genome sequencing (WGS).

These amplified products are then sequenced with the use of one of the various sequencing technologies (e.g., Illumina’s sequencing by synthesis, Life Technologies’ sequencing by ligation, or Ion semiconductor sequencing) to generate millions of short sequence reads. These sequences are then processed bioinformatically. First, they are aligned to a reference genome (assembly) and then compared for similarities and differences at each target position. A list of variants (or differences in the sequence) is then generated, which is then filtered further, to determine the significance of each of the variant. Common filters include rare or previously unreported variants, variants that lead to altered function of the protein, and variants previously reported to cause disease [17]. More recently, algorithms that include the phenotypic information in the variant analysis have been developed that aid the clinician/researcher in narrowing down the candidate gene list [18, 19].

### Next-generation sequencing in the clinical setting

#### Targeted vs whole exome sequencing

One of the biggest challenges clinicians are facing is deciding between using targeted versus whole exome sequencing. As the cost of sequencing continues to decrease, WES appears to be a more cost-effective approach. However, there are certain considerations before embarking on one over the other.

Although exomes are supposed to cover the protein-coding regions of the genome, the overall coverage tends to be between 85–95 % only. This means that a particular gene of interest with respect to a specific phenotype may not be covered, either completely or partially. Reasons include poorly performing capture probes due to high GC content, sequence homology, or repetitive sequences. A targeted approach, on the other hand, has a much higher or even complete coverage of all the phenotype-specific genes by filling in the gaps with complementary technologies such as Sanger sequencing or long range PCR. For example, 4 of the 73 genes in a hearing loss panel are inadequately covered on WES but are completely covered in a targeted panel [20].

Besides offering a more comprehensive coverage of the “known” phenotype-specific genes, a targeted approach also allows for deeper coverage of these genes compared to WES, which provides greater confidence in the variants detected. However, both are still prone to sequencing artifacts, and Sanger sequencing of candidate variants is recommended in both approaches before returning the results to the patients [20].

Lastly, laboratories that offer targeted testing may have expert knowledge for the given phenotype and may be in a better position to prioritize variants detected through NGS. They may also be able to recommend specific evaluations to determine the significance of certain variants; for example, temporal bone evaluation for *SLC26A4* and otoacoustic emission testing for *OTOF* when variants are detected on a targeted hearing loss panel [20].

#### Indications and clinical usefulness

NGS is currently indicated for the detection of rare variants in patients with a phenotype suspected to be due to a Mendelian disease. This is done either after single gene testing for candidates has returned negative, or as first line, if there exists genetic heterogeneity where multi-gene Sanger sequencing would be costly and time consuming. Diseases could either include a specific phenotype such as cardiomyopathy, deafness, retinitis pigmentosa, intellectual disability, or be part of a multiple congenital anomaly spectrum (where two or more organs are affected) [21].

The clinical usefulness of performing NGS in the clinical setting varies for different disorders. In the majority of cases, the finding does not alter the clinical management, treatment or prognosis. However, it does allow an end to an expensive and stressful diagnostic odyssey. Reaching a molecular diagnosis allows the clinician to allay the guilt that parents face in the absence of a firm diagnosis [13, 17] and helps them to accept the child’s condition.

Identification of the causative variant allows for gene-specific prognostication [22, 23], based on cases reported in the literature and, in some cases, family support groups. It can also allow for anticipatory management of other comorbidities that an individual may be susceptible to. These include assessing other organs (such as performing blood tests and imaging to assess the heart and kidneys) for possible complications. In some cases, it may redirect therapy to treat the underlying genetic etiology as illustrated by the case of a 15-month-old child with clinical presentation mimicking Crohn’s disease. WES revealed a pathogenic variant in *XIAP*, affecting the proinflammatory response and bacterial sensing, predisposing the individual to developing hemophagocytosis. Based on these findings, the child was successfully treated with hematopoietic bone marrow transplantation which cured him of his gastrointestinal disease as well [24].

More importantly, it facilitates genetic counseling and allows for more accurate estimates of recurrence risk in the family. Identification of the molecular etiology allows the clinician to guide subsequent pregnancies, either through prenatal diagnostics or preimplantation genetic diagnosis. In some situations, it also allows for identification of other at-risk family members and any available treatment can be instituted in the presymptomatic phase [17]. For example, identification of mutation in a gene causing long QT syndrome in the proband can allow identification of other at-risk family members, who can then have regular Holter monitoring and, in some instances, implantation of a cardiac pacemaker before a catastrophic event occurs [25, 26].

Lastly, as our understanding of the molecular pathways and gene-gene interactions improves, it is possible that targeted molecular therapy may be available for a specific genetic mutation that helps to ameliorate the patient’s symptoms. For example, in individuals with vascular malformations, somatic mutations in the *AKT3-PI3K-mTOR* pathway have been identified. Some of these patients have been successfully treated with mTOR-inhibitors such as rapamycin [27, 28]. Our ability to offer such targeted therapy will only improve with time as more high-throughput drug screening methods are being deployed.

#### Gathering information

The process of ordering NGS starts with gathering a detailed family history to determine if there are individuals with similar or related phenotypes within the same family, as well as to assess for possible inheritance pattern. For example, multiple individuals in the same generation and/or history of consanguinity will suggest a recessive pattern, while having affected individuals in each generation would suggest a dominant pattern [4].

The next step is to perform detailed phenotyping of the affected individual(s). These could include evaluations from other subspecialists and/or performing biochemical and/or radiological tests. For example, a child with a limb anomaly would benefit from cardiac, gastrointestinal, renal, and skeletal evaluations. With the phenotype and pedigree information, a systematic review of literature or syndrome database should be performed to exclude rare but established syndromes [17]. This can then guide the clinician on which gene(s) to test. In cases of genetic heterogeneity, targeted NGS may be the preferred approach. On the other hand, if the disease mechanism is unknown, WES may be the test of choice.

#### Pretest counseling and informed consent

It is imperative that a patient and his/her family are counseled appropriately by a healthcare professional (such as a clinical geneticist or genetic counselor) who is aware of the nuances of the test. It is important to maintain realistic expectations for the patient and his/her family, as it is possible that the testing may not return any positive results. The diagnostic yield would differ on the test being performed. In a targeted approach, it is possible that the causal variant is in a gene that was not in the subset of genes or regions that were targeted. On the other hand, as the coverage in WES tends to be ~85–95 %, it is possible that the causal variant lies in the region that was either poorly covered or resides outside the protein-coding region of the gene (such as the promoter or regulatory region) [17]. The sensitivity and limitations of the specific NGS test must be clearly communicated.

It is also important to emphasize to the families that a positive result may not change treatment or management decisions, and hence, may not alter the prognosis or outcome for the affected individual [17]. But it is also possible that the test may identify targets that may be amenable to certain medications. This has been the case in the field of oncology, where specific molecular targets are used to treat certain subtypes of cancer.

Cost remains an important consideration as clinical targeted NGS or WES can cost between USD 2000 to 10,000 and USD 5000 to 15,000, respectively [17, 20]. Insurance companies may not approve such costly tests, and in self-payer healthcare systems (such as Singapore) where the individual has to bear the cost, this may be prohibitively expensive.

Lastly, the patient should be advised that variants which are not related to the primary phenotype, also referred to as secondary or incidental findings (IFs), may be detected. Detection of IFs is more common with WES and WGS and may be less of an issue with targeted panels. This may have implications not only for the individual, but also his/her family members [17, 20]. For example, WES on a patient with intellectual disability may detect pathogenic variants in *BRCA1*. This mutation could be inherited from the parents, which would mean that the affected parent (and his/her sibling) is at risk of developing cancer and would require surveillance and monitoring. This family may or may not be prepared to receive such information and this should be discussed during the informed consent process. While genetic nondiscrimination act (GINA 2008) protects individuals against discrimination in the USA, similar laws are lacking in other countries [29]. Hence, individuals with a genetic diagnosis, especially those with incidental findings, may face discrimination at work or be denied medical insurance without any avenue for legal redress.

In 2013, the American College of Medical Genetics and Genomics (ACMG) recommended that laboratories performing WES should report on incidental findings detected in a minimum set of 56 genes. These 56 genes represent disorders that are highly medically actionable and include *BRCA1* and *BRCA2* [30]. In 2015, ACMG updated the recommendations allowing patients and/or parents to opt out of such analysis [31]. On the other hand, the European Society of Human Genetics (ESHG) advises for prudent use of WGS-based testing and to restrict testing to regions of the genome linked to the patient’s phenotype in order to avoid detection of such incidental findings [32]. Even for exome or whole genome sequencing, it is possible to mask the results of specific genes with a targeted workflow so that they are excluded from subsequent analysis for variant identification. Thus, physicians would not have to make the hard decisions about the reporting of such incidental findings.

#### Interpretation of results

On average, ~60,000 to 100,000 variants are detected on WES. These variants can be broadly classified into pathogenic, benign, or variants of uncertain significance (VUS). Pathogenic variants are defined as those variants that adversely alter protein function and have either been reported previously in other affected individuals or have been shown to affect protein function in cellular or animal models. These include variants such as nonsense, frameshift, splicing, small insertion-deletions (indels), or nonsynonymous missense variants. Benign variants, also known as polymorphisms, are variants that exist in a significant proportion of the population, including healthy individuals, and account for majority of the variants detected on NGS testing. These include synonymous missense variants, intronic, or intergenic variants [33].

VUS are variants that could possibly affect protein function based on in silico software (such as Polyphen-2 [34] and SIFT [35, 36]) or other similar parameters, but either have not been described in other individuals (affected or unaffected) or do not have any functional analysis in other model systems [33]. As there are genomic variations across different populations and ethnic groups, a database of common variants from healthy individuals is imperative in understanding the significance of a given variant. Such databases include dbSNP, 1000 Genomes project, Exome Aggregation Consortium (ExAC), and Exome Sequencing Project (ESP), but certain ethnicities are underrepresented in these databases. Interpretation of VUS in such instances requires segregation analysis (by analyzing the variant in other family members)—presence of the variant in affected but not in unaffected family members adds further evidence to the possible causal relationship of a given variant. An alternative approach includes testing the variant in cellular and/or animal models, but this is beyond the realms of a clinic or clinical laboratory and may be best addressed through a research laboratory.

There is a wide range of possible outcomes with NGS testing. Using WES, a single pathogenic variant that is likely to be the cause of the disease can be detected about 20–36 % of the time [14–16, 37, 38]. For the remainder of the cases, it is possible to either find multiple candidate variants or none at all. In the event that multiple candidate variants are detected, segregation analysis and/or functional analysis would help to determine the molecular etiology. If no candidate variants are found, possibilities include poor coverage or the mutation residing outside the protein-coding region of the gene or the defect is not due to a simple nucleotide change in a single gene.

#### Delivery of results

Clinicians should review the results of NGS and correlate the findings with the relevant medical information. When a pathogenic variant is detected in a gene that explains the patient’s clinical phenotype, the clinician should review the results as well as relevant clinical information, including inheritance, prognosis, complications, or management, with the patient and his/her family. Testing of at-risk individuals should be offered, when possible.

On other occasions, finding of a particular variant may prompt the clinician to look for additional history or perform tests which may or may not support the conclusion of the NGS test report. For example, in a patient with chronic diarrhea, finding biallelic mutations in *TTC37* may prompt the clinician to review the hair for specific features of trichorrhexis nodosa, suggestive of a diagnosis of tricho-hepato-enteric syndrome [39]. On the other hand, if more than one candidate variant is detected, the clinician will need to perform further evaluation(s) to determine which of the variant is causing the phenotype.

Lastly, if the test results are negative, reasons for this should be discussed with the patient (as discussed in the pretest counseling). In addition, as our understanding of the human genome improves and more similar cases are reported, there may be a possibility of associating the patient’s phenotype to a newly described genetic syndrome. This requires reanalysis of the both clinical reports and genetic data at regular intervals. However, regulations regarding who and how to perform reanalysis are currently lacking and remain a challenge for the near future.

### Future directions

Although disease-targeted testing may remain useful in the short term, as our knowledge improves, addition of newly identified genes to the panel (which has to be synthesized according to customized order) may take time, may be laborious and may not be cost-effective for the laboratory. Many laboratories have now shifted to performing WES and limiting the analysis to genes associated with phenotype and filling up the gaps with Sanger sequencing. Although the cost may be 2–5 times higher than targeted panel sequencing (which can go as high as USD 3000 depending on the number of genes in the panel), it allows for reanalysis of the data when new gene associations are made. In addition, some commercial companies have developed kits that capture only medically relevant regions of the genomes. These kits eliminate the risk of finding variants in genes whose function is unknown [20].

Currently, clinicians order NGS testing based on detailed phenotyping and after excluding single candidate genes—also referred to as a “phenotype first” approach. As NGS testing becomes more easily available, there will be a tendency for clinicians to perform the testing first and then assess the patient’s phenotype to match the genotype—referred to as “genotype first” approach [40].

It is also possible that in the not so distant future, a relatively healthy individual may perform WES first and then consult a clinician for interpretation of the findings and subsequent evaluations. It may not be long before newborns are screened for inherited disorders using WES. However, there are many ethical, medical, and logistical challenges that need to be addressed before this becomes common practice [20, 41].

Finally, there have been recent reports on the role of somatic mutations in Mendelian diseases. This has been advanced by the ability to perform deep-targeted NGS to detect low level mutations in patients with Mendelian disorders, majority of which would have been missed by conventional testing [42]. As our understanding of the role of somatic mutations in other human diseases increases, deep-targeted NGS may be the test of choice in these disorders.

## Conclusion

NGS is a useful diagnostic test for the majority of Mendelian disorders and is gaining acceptance in the medical community. However, there remain certain key challenges that need to be addressed before NGS testing becomes part of routine clinical practice.
